# Mogroside II_E_ Inhibits Digestive Enzymes *via* Suppression of Interleukin 9/Interleukin 9 Receptor Signalling in Acute Pancreatitis

**DOI:** 10.3389/fphar.2020.00859

**Published:** 2020-06-10

**Authors:** Juan Xiao, Kai Huang, Houmin Lin, Zhijia Xia, Jing Zhang, Dianpeng Li, Junfei Jin

**Affiliations:** ^1^Laboratory of Hepatobiliary and Pancreatic Surgery, the Affiliated Hospital of Guilin Medical University, Guilin, China; ^2^China-USA Lipids in Health and Disease Research Center, Guilin Medical University, Guilin, China; ^3^Guangxi Key Laboratory of Molecular Medicine in Liver Injury and Repair, Guilin Medical University, Guilin, China; ^4^Guangxi Key Laboratory of Functional Phytochemicals Research and Utilization, Guangxi Institute of Botany, Guangxi Zhuang Autonomous Region and Chinese Academy of Sciences, Guilin, China; ^5^Guangxi Key Laboratory of Sphingolipid Metabolism (Incubated), Guilin Medical University, Guilin, China

**Keywords:** acute pancreatitis, mogroside II_E_, interleukin 9, interleukin 9 receptor, trypsin, calcium, impaired autophagy

## Abstract

The incidence of pancreatitis (AP) is increasing and there is no specific treatment available. Intracellular digestive enzyme activation is a key event in the pathogenesis of AP downstream of cytosolic calcium overload and impaired autophagy. *Siraitia grosvenorii* (Swingle) was used in Traditional Chinese Medicine to reduce inflammation and facilitate bowel movement. The bioactive components of this plant show hypolipedimic, antidiabetic, antifibrotic activity and have been used against pancreatic cancer. Here, we examined whether mogroside II_E_, a major bioactive component of unripe *S. grosvenorii* fruit, can protect against AP. We found that mogroside II_E_ decreased the activity of trypsin and cathepsin B induced by cerulein plus lipopolysaccharide (LPS) in the pancreatic acinar cell line AR42J and primary acinar cells in a dose- and time-dependent manner. Mogroside II_E_ treatment decreased the levels of serum lipase and serum amylase in mice injected with cerulein plus LPS without influencing inflammation significantly. A multi-cytokine array revealed that mogroside II_E_ decreased the level of interleukin 9 (IL-9) in AP mice. Exogenous IL-9 eliminated the mogroside II_E_ induced reduction of trypsin and cathepsin B activity and reversed the inhibition of cytosolic calcium and modulation of autophagy mediated by mogroside II_E_. An IL-9 receptor antibody neutralized the effect of IL-9, restoring mogroside II_E_ activity. The mogroside II_E_ targeted IL-9 may partially arise from Th9 cells. Taken together, we provide experimental evidence that mogroside II_E_ ameliorates AP in cell models and mice through downregulation of the IL-9/IL-9 receptor pathway.

## Introduction

Acute pancreatitis (AP) is a common inflammatory disease without specific treatment (Moggia et al., 2017; Lee and Papachristou, 2019). In Traditional Chinese Medicine (TCM) bitter purgatory decoctions improving gastrointestinal motility and blood circulation and reducing inflammatory injury are used to ameliorate AP (Li et al., 2019; Zhang et al., 2019). *Siraitia grosvenorii* (Swingle) is used in TCM as a general anti-inflammatory agent as well as bowel movement facilitator and a laxative (Zhang et al., 2020). The active molecules from *S. grosvenorii* (Swingle) have also been regarded as hypolipedimic, anti-fibrotic and anti-diabetic (Chen et al., 2011; Tao et al., 2017b), which make them relevant candidate drugs against pancreatic diseases. In particular, *S. grosvenorii* (Swingle) bioactive components showed benefits in pancreatitic cancer models (Liu et al., 2016). Thus, despite not being part of anti-AP TCM decoctions, we decided to investigate if mogroside II_E_, a major bitter taste bioactive component of unripe *S. grosvenorii* (Swingle) fruit (Wang et al., 2014) can protect against AP.

Intrapancreatic trypsinogen activation is central in the pathogenesis of AP. It is believed to be initiated by calcium overload or impaired autophagy (Xiao et al., 2016). Independent of trypsin, nuclear factor-k-gene binding (NFkB) pathway activation induce cytokine release which lead to local or systematic inflammation in the development of AP (Saluja et al., 2019). TNFα and IL-1β are secreted in the early stage of AP (Kim et al., 2020). IL-1β initiates a cytokine cascade that results in systemic inflammatory response syndrome in AP (Norman, 1998). IL-10 is induced in early AP, but it is an anti-inflammatory cytokine (Gloor et al., 1998). IL-6 levels which could be induced by TNFα and IL-1β, are elevated in pancreatitis and serve as markers of the severity of pancreatitis (Staubli et al., 2015; Li et al., 2018). Platelet-activating factor directly causes pancreatitis (Jakkampudi et al., 2016). Chemokines, such as IL-8, MCP-1 and regulated upon activation, normal T cell expressed and presumably secreted (RANTES), are pro-inflammatory mediators (Bhatia, 2005). A cytokine storm results in multiple organ dysfunction, which leads to treatment failure in severe AP patients. However, the roles of other cytokines in AP are not fully characterized.

IL-9 is mainly secreted from T cells, and it is involved in type 2 immunity and regulates allergic inflammation (Chakraborty et al., 2019). IL-9 is approximately 14 kD, and it may be glycosylated. IL-9 recognizes and binds to IL-9 receptor (IL-9R) in target cells, which is a member of the type I hematopoietin receptor superfamily and exhibits high affinity for IL-9 (Chakraborty et al., 2019). However, the relationship between IL-9 and AP are barely understood.

In our study we examined the effect of mogroside II_E_ on molecular markers of AP, focusing on pancreatic enzyme activity, cell calcium concentration, and systemic cytokine release. Out of 23 cytokines tested, we found a very significant reduction in IL-9 in the presence of mogroside II_E_. Hence, a large part of our work was dedicated to elucidate the effect of mogroside II_E_ on IL-9 pathway and its role in AP.

## Materials and Methods

### Reagents

Magoside II_E_ (HPLC ≥98%) was purchased from Chengdu Man Si Te company (Chengdu, Sichuan, China) (http://www.cdmust.com/). Cerulein and 3-MA were purchased from MCE. LPS was purchased from Sigma. IL-9 was purchased from Origene. The IL-9 receptor antibody was purchased from SAB. β-actin and p62 antibodies were purchased from PTG. The LC3 antibody was purchased from Sigma. Fura-2 AM was purchased from Sigma. Fluro-4 AM was purchased from Solarbio.

### Cell Culture and Primary Acinar Cell Isolation

AR42J cells (exocrine pancreatic tumor cells, ATCC) were cultured in Dulbecco's modified Eagle's medium (DMEM, Gibco) containing 10% fetal bovine serum (FBS, Gibco) and 1% penicillin–streptomycin (Solarbio). All cultures were maintained in a 37°C incubator with 5% CO_2_. Pancreas tissue was separated from C57BL/6 mice. Collagenase was used to digest pancreatic acinar cells. After filtration, primary pancreatic acinar cells were cultured in F12K (Gibco) supplemented with 10% FBS and used for subsequent experiments. The Ethical Committee on Animal Experiments at Guilin Medical University approved all animal care and experimental procedures, which were performed in accordance with the National Institutes of Health Guide for the Care and Use of Laboratory Animals (NIH Publications No. 8023, revised 1978).

### Compound Treatment of Pancreatic Acinar Cells

AR42J cells or primary acinar cells were seeded in 12-well plates. Cells were treated with solvent (Control) or 200 nM cerulein plus 10 ng/ml LPS (C + L) for 6 h. Mogroside II_E_ (M) (20 μM) was added for the indicated durations, or different concentrations of M were added for 6 h with C + L. IL-9 (10 ng/ml) was added to cells in the presence of C + L + M (20 μM) (C + L + M + IL9) for 6 h. The autophagy inhibitor 3-MA (2 mM) was added to cells with C + L or C + L + IL9 for 6 h. The IL-9 receptor antibody (2.5 μg/ml) was added to cells with C + L + M + IL9 for 6 h.

### Mogroside II_E_ and IL-9 Treatment in Mouse

Female C57BL/6 mice, 6–8 weeks old, 20 g, were purchased from SLAC (Changsha, Hunan). Mice were housed in clean environment and were deprived from food before any treatments (n = 6 in each group). Control group: mice were intraperitoneally injected with saline seven times at one-hour intervals, and solvent was given after the first saline injection. C + L group: cerulein (50 μg/kg) were intraperitoneally injected seven times, and LPS (10 mg/kg) was injected once after the final cerulein injection. C + L + Mlong group: cerulein and LPS treatments were the same as the C + L group, mogroside II_E_ (10 mg/kg) was injected once after the first injection of cerulein. C + L + Mshort group: similar to the C + L + Mlong group but mogroside II_E_ was given after the final cerulein injection. C + L + Mlong + IL9 group: similar to the C + L + Mlong group and IL-9 (10 mg/kg) was intraperitoneally injected after the first injection of cerulein. Mice were sacrificed 6 h after the final injection of saline or cerulein. Sera were collected to measure lipase and amylase. Pancreas tissues were lysed to measure the activity of trypsin and cathepsin B.

### Th9 Cell Measurement

After mogroside II_E_ and IL-9 treatment (described above, n = 3 in each group), mice were sacrificed. Spleens were ground. The homogenate was filtered using a 200-mesh cell sieve and centrifuged. Cell pellets were washed with PBS. After centrifugation, the cell pellet was incubated with red blood cell lysis buffer (Pharmingen, BD Biosciences, NJ, USA). Each sample was adjusted to a concentration of 1 × 10^6^ cells/well. These cells were incubated with specific antibodies, including APC-CD4 (Pharmingen, BD Biosciences, NJ, USA) and PECy7-CD8 (Pharmingen, BD Biosciences, NJ, USA) and fixed and stained with a PE-IL9 antibody (Pharmingen, BD Biosciences, NJ, USA). Th9 cells are CD4^+^/CD8^−^/IL9^+^, and flow cytometry was used for analysis.

### Trypsin Activity Assay

Pancreatic acinar cells were lysed and incubated with butoxycarbonyl-Gln-Ala- Arg-7-amido-4-methylcou-marin hydrochloride (Sigma) in trypsin reaction buffer (10 mM Tris, 20 mM CaCl2, pH 7.4) at 37°C for 30 min. The fluorescence intensity of the reaction solution, which reflects trypsin activity, was measured (excitation: 380 nm, emission: 450 nm) in a fluorescence microplate reader. Purified trypsin protein was added to the reaction mixture separately as the standard. In some experiments, trypsinogen activation was detected using the trypsin fluorescent substrate Rhodamine 110, bis-(CBZ-L-isoleucyl-L-prolyl-L-arginine amide) (Sigma). Briefly, 50 μM of this molecule was added to cells for 20 min, and cells were imaged with microscopy. The fluorescence intensity reflecting trypsin activity was qualified using ImageJ software.

### Cathepsin B Activity Assay

Cells or tissue were lysed. Cathepsin B activity was measured according to the Enzymatic Assay of Cathepsin B protocol provided by Sigma, Inc. Briefly, cell lysates were incubated with cathepsin B substrate Nα-CBZ-Arg-Arg-7-amido-4-methylcoumarin at 37°C, and the fluorescence intensity was determined at 440 nm in a fluorescence microplate reader under excitation at 348 nm. Purified cathepsin B protein was added to the reaction mixture separately as the standard.

### Calcium Measurement

Fura-2 AM was added to acinar cells according to the protocol provided by the manufacturer. Once indicated compounds were added, fluorescence was detected with spectrophotometer (Molecular Devices) with excitation wavelengths of 340 and 380 nm and emission at 505 nm. Dynamic changes in cytosolic calcium were monitored by using the Fura-2 340/380 nm fluorescence ratio. Endpoint calcium was also detected with Fluo-4 AM using a microscope (Themo EVOS M5000). After the indicated treatments, acinar cells were stained with Fluo-4 AM, imaged and quantified.

### Serum Lipase and Amylase Measurements

Serum lipase was measured using an automatic-chemistry analyser (Roche, Cobas 8000).

### HE Staining and Pathological Analysis

Formalin-fixed pancreas samples were processed, and 4-µm thick paraffin sections were stained with hematoxylin and eosin (H&E). In this study, histopathological changes, including edema, necrosis and infiltration of inflammation cells were qualified according to the criteria described in **Figure S3** (Ou et al., 2015). The final score was calculated as the sum of the above-mentioned three parameters. All sections were evaluated by two independent pathologists unaware of the study target.

### Multi-Cytokine Array

Luminex Mouse Cytokine/Chemokine Magnetic Bead Panel (MCYTOMAG-70K-23) was used, and cytokines were detected according to the manufacturer's instructions.

### Statistical Analysis

SPSS 19.0 (SPSS Inc., Chicago, IL) was used for statistical analyses. One-way analysis of variance or Student's t test was used to evaluate the statistical significance.

## Results

### Mogroside II_E_ Inhibits Trypsin Activity of Unstimulated Cells

**Figure 1:**
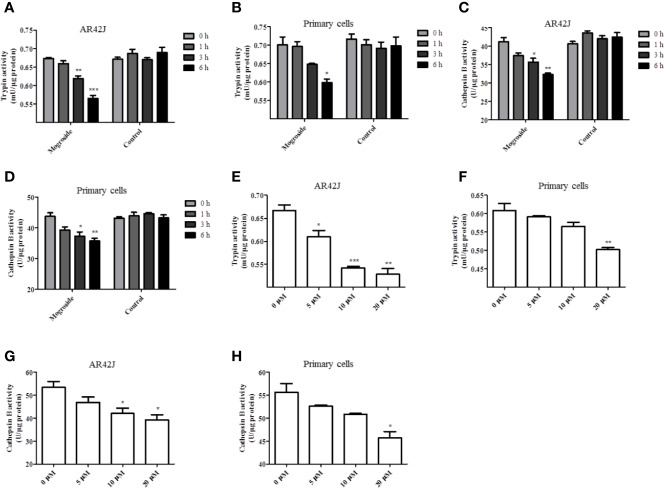
Mogroside II_E_ inhibits trypsin activity in a time- and dose-dependent manner. AR42J cells **(A**, **C)** or primary pancreatic cells **(B**, **D)** were treated with 20 μM mogroside II_E_ or solvent (Control) for 0, 1, 3 and 6 h. Cells were lysed, and cell lysates were subjected to trypsin **(A, B)** or cathepsin B **(C, D)** activity assays. Vs. 0 h time point, ^*^P < 0.05, ^**^P < 0.01. AR42J cells **(E**, **G)** or primary pancreatic cells **(F**, **H)** were treated with 0, 5, 10, 20 μM mogroside II_E_ for 6 h. Cells were lysed, and cell lysates were subjected to trypsin **(E, F)** or cathepsin B **(G, H)** activity assays. Vs. 0 μM, ^*^P < 0.05, ^**^P < 0.01, ^***^P < 0.001.

The effect of mogroside II_E_ on trypsin activity was investigated first. AR42J cells and primary pancreatic acinar cells were cultured, and mogroside II_E_ was added for 1, 3, or 6 h. The results showed that the compound inhibited trypsin in a time-dependent manner (**Figures 1A, B**). Since cathepsin B is implicated in trypsinogen activation, we have measured cathepsin B activity. The results showed that mogroside II_E_ also inhibited cathepsin B activation (**Figures 1C, D**). Notably, enzyme activity did not change significantly with solvent treatment for different durations. Similar dose-dependent effects of mogroside II_E_ on trypsin and cathepsin B activities were observed in AR42J cells and primary pancreatic acinar cells (**Figures 1E–H**).

Taken together, mogroside II_E_ inhibited trypsin and cathepsin B activity. According to the time effect experiment, 6 h was chosen for the next investigation.

### Mogroside II_E_ Inhibits Cerulein Plus LPS-Induced Trypsinogen Activation in Pancreatic Acinar Cells

**Figure 2:**
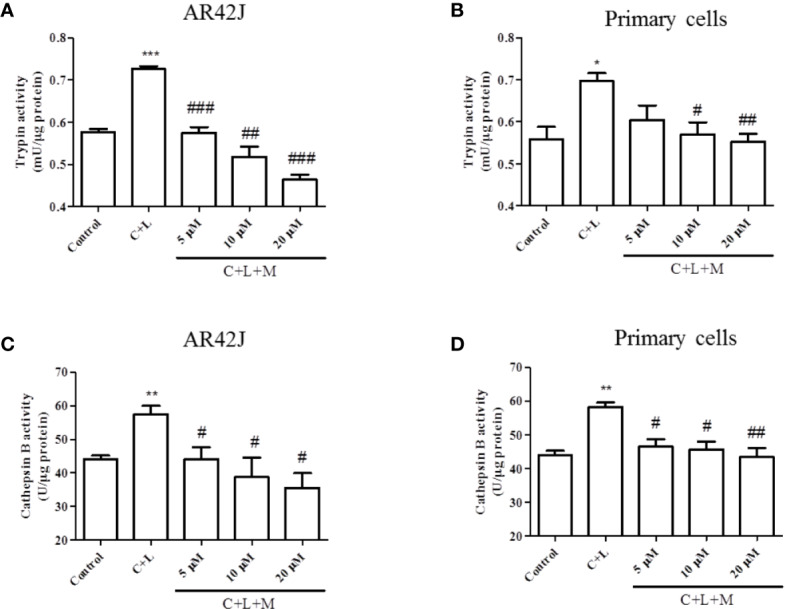
Mogroside II_E_ inhibits trypsin and cathepsin B induced by cerulein in pancreatic acinar cells. AR42J cells **(A**, **C)** or primary pancreatic cells **(B**, **D)** were treated with solvent (Control), 200 nM cerulein plus 10 ng/ml LPS (C + L), C + L with 5, 10, 20 μM mogroside II_E_ (C + L + M) for 6 h. Cells were lysed, and cell lysates were subjected to trypsin **(A**, **B)** or cathepsin B **(C**, **D)** activity assays. C + L vs. Control, ^*^P < 0.05, ^**^P <0.01, ^***^P < 0.001. *C* + L + M vs. C + L, ^#^*P <* 0.05, ^##^*P <* 0.01, ^###^*P <* 0.001.

The above-described results showed that mogroside II_E_ decreased the background level of trypsin activity. Therefore, we examined whether it showed similar effects in pancreatic acinar cells under AP inducer, cerulein plus lipopolysaccharide (LPS) treatment. Mogroside II_E_ reduced the activity of trypsin and cathepsin B (**Figure 2**). Notably, mogroside II_E_ at low concentrations (5 or 10 μM) significantly decreased cerulein plus LPS-induced trypsin activation without showing obvious inhibition on the background level of trypsin activity (**Figures 2A, B**). The changes in cathepsin B matched the fluctuations in trypsin activity (**Figures 2C, D**).

In summary, mogroside II_E_ inhibited cerulein plus LPS-induced trypsin activation in pancreatic acinar cells, which may occur *via* inhibition of cathepsin B.

### Mogroside II_E_ Inhibits Trypsinogen Activation in AP Mouse

**Figure 3:**
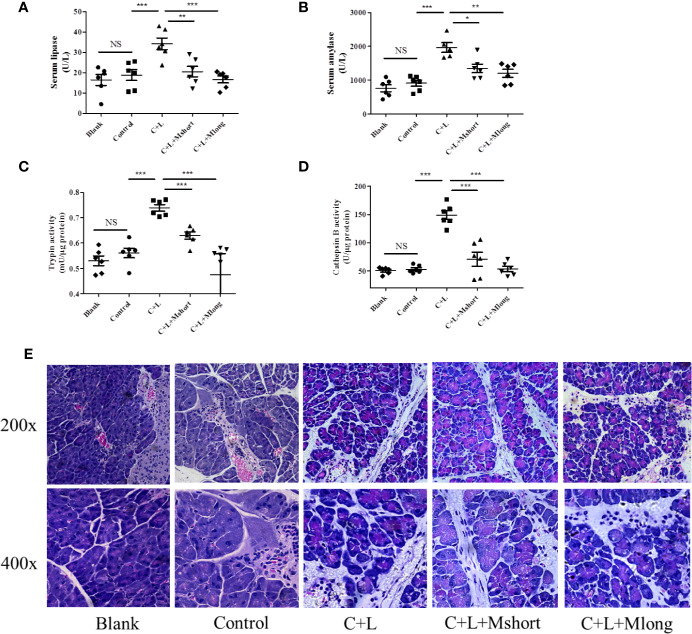
Mogroside II_E_ inhibits trypsin in AP mice. Female C57BL/6 mice were randomly divided into five groups (n = 6): Blank, Control, C + L, C + L + Mshort, C + L + Mlong. After the indicated treatments, mice were sacrificed. Sera were collected to measure the serum level of lipase **(A)** and amylase **(B)**. Part of the pancreas was lysed to analyse trypsin **(C)** and cathepsin B **(D)**. The other part of pancreas was fixed and used for HE staining **(E)**. For all of the graphs, NS, non-significant, ^*^P < 0.05, ^**^P < 0.001, ^***^P < 0.001.

To validate the effect of mogroside II_E_ on trypsin, an AP mouse model was constructed using intraperitoneal injections of cerulein plus LPS. Trypsin activation induces lipase activation and amylase release from pancreatic acincar. Therefore, serum lipase and amylase reflect the activity of trypsin to some extent. The time course of serum lipase and amylase in the animal model were studied. The results showed that digestive enzymes reached the maximum 3 h after the final injection of cerulein (**Figure S1**). Mogroside II_E_ was injected early or later, which indicated the long or short time treatment of the compound. After compound treatment, mice were sacrificed 1, 3 and 6 h after the final cerulein injection. The results showed that mogroside II_E_ did not show significant inhibition of digestive enzymes at 1 or 3 h (**Figure S2**). Mogroside II_E_ significantly reduced serum lipase and amylase in AP mice at 6 h (**Figures 3A, B**). Enzyme activity in pancreas tissue from the mice was detected at 6 h. Trypsin and cathepsin B activation were inhibited in pancreas from AP mice with mogroside II_E_ (**Figures 3C, D**), which is consistent with the serum enzyme analysis. Early injection or long time treatment of mogroside II_E_ showed better effects on these two enzymes (**Figures 3C, D**). These results suggest that mogroside II_E_ inhibited the activation of trypsin and cathepsin B in AP mice, which is consistent with the phenomena *in vitro*.

### Mogroside II_E_ Did Not Affect the Pathology of the Pancreas From AP Mouse

Mogroside II_E_ inhibited trypsin activity and digestive enzymes in serum. To investigate the roles of this molecular in AP further, pathological changes were detected in AP mice with mogroside II_E_ treatment. Pathological evaluation was performed using HE staining. Obvious edema and immune cell infiltration were observed in cerulein plus LPS-injected mice compared to the control. Short and long term treatment with mogroside II_E_ did not significantly reverse the pathological changes in AP mice (**Figure 3E** and **Figure S3**). Edema and interstitial immune cells were still observed (**Figure 3E** and **Figure S3**). Taken together, mogroside II_E_ showed little function in ameliorating AP pathology.

### Mogroside II_E_ Inhibits IL-9 Release

**Figure 4:**
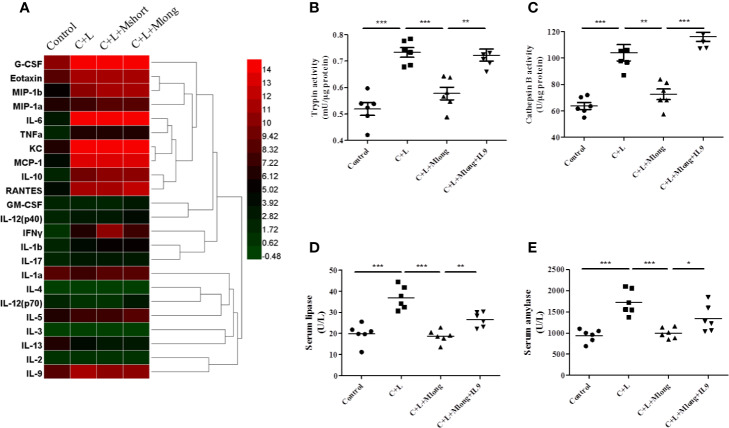
Mogroside II_E_ inhibition of digestive enzymes is dependent on IL-9 in AP mice. Mice (n = 6) in Control group, C + L group, C + L + Mshort group, C + L + Mlong group were sacrificed, and serum and pancreas tissues were collected. **(A)** Serum was subjected to multi-cytokine array, and a heatmap of changes in serum cytokines from mice under indicated treatments was shown. Serum lipase **(D)** and amylase **(E)** were analyzed. ^*^P < 0.05, ^**^ *P <* 0.001, ^***^P < 0.001. Pancreas tissues were lysed, and trypsin **(B)** and cathepsin B **(C)** were detected.

Inflammation presents independent of trypsinogen activation and contributes greatly to the high mortality in AP. Therefore, the effect of mogroside II_E_ on inflammation in AP was investigated. AP mice were treated with or without Mogroside II_E_ and inflammatory mediators in serum were detected using a cytokine array containing 23 cytokines (**Figure 4A** and **Table 1**).

**Table 1:** Cytokine array results of sera from AP mice with indicated treatments (Mean ± SD, pg/ml).

	Control	C + L	C + L + Mshort	C + L + Mlong
G-CSF	1,382.3 ± 406.7	66,089.3 ± 11,219.2***	64,803.8 ± 8,643.3	62,867.3 ± 11,321.5
Eotaxin	534.3 ± 198.1	2,192.5 ± 652.7***	2,791.8 ± 561	3,036.7 ± 334
GM-CSF	3.5 ± 1.2	6.7 ± 3	5.5 ± 3.4	8.6 ± 9.2
IFNγ	2.1 ± 0.7	96.8 ± 76.5	1,468.4 ± 3,244	199.4 ± 330
IL-1a	536.8 ± 203.7	262.3 ± 95.7	453.6 ± 567.1	449.4 ± 316.9
IL-1b	3.7 ± 0.8	32 ± 6*	58.8 ± 38.6	33.2 ± 24.8
IL-2	2.1 ± 0.5	2.9 ± 0.8	2.3 ± 0.9	2.6 ± 0.6
IL-3	1.2 ± 0.2	1.1 ± 0.2	0.9 ± 0.1	1.1 ± 0.2
IL-4	0.9 ± 0.1	0.8 ± 0.1	0.7 ± 0.1	1.2 ± 0.9
IL-5	140.5 ± 110.1	150.1 ± 103.2	165.7 ± 187.9	412.5 ± 792.7
IL-6	13.3 ± 11.1	19,217.5 ± 8,403.7***	17,858 ± 9,329.8	23,864.8 ± 6,428.2
IL-9	602.1 ± 342	2,916.7 ± 1,206.2***	1,254.3 ± 603.6^##^	10 ± 303.2^##^
IL-10	1,382.3 ± 406.19	66,089.3 ± 11,219.14	64,803.8 ± 8,643.2	62,867.3 ± 11,321.17
IL-12 (p40)	4.3 ± 1.2	14.4 ± 7.6	8.7 ± 5.4	18.6 ± 14.9
IL-12 (p70)	5.4 ± 4.5	3.2 ± 1	1.7 ± 0.3	8.8 ± 15.8
IL-13	124.3 ± 33.9	15 ± 3.9***	9.8 ± 1.5	11.9 ± 1.4
IL-17	3.6 ± 0.8	8.1 ± 4.2	10.6 ± 7.9	7.8 ± 5.4
KC	80.8 ± 21.3	21,097.8 ± 7,237.5***	27,249.8 ± 6,747.6	28,312.5 ± 3,154.4^#^
MCP-1	27.6 ± 14	9,247.3 ± 4,598*	13,615.7 ± 9,766	15,209.5 ± 6,064.5
MIP-1a	76.2 ± 40.8	281.4 ± 65.6**	357.1 ± 112.2	471.9 ± 160.9^##^
MIP-1b	54.4 ± 16.3	1,749 ± 642.8*	2,637.7 ± 1,159.4	3,486 ± 1,707^#^
RANTES	15 ± 11.7	2,679.8 ± 1,011*	2,795.3 ± 1,284.5	4,089.7 ± 2,961.6
TNFα	3.8 ± 1	90.5 ± 66.1**	94.8 ± 49.6	87.5 ± 37.4

C + L vs. Control, ^*^P < 0.05, ^**^P < 0.01, ^***^P <0.001.

C + L + Mshort or C + L + Mlong vs. C + L, ^#^P < 0.05, ^##^P < 0.01.

Some cytokines, including granulocyte colony-stimulating factor (G-CSF), eotaxin, IL-1β, IL-6, IL-9, keratinocyte cytokine (KC), monocyte chemotactic protein 1 (MCP-1), macrophage inflammatory protein 1α (MIP-1α), and macrophage inflammatory protein 1β (MIP-1β), RANTES and TNFα, are increased significantly in AP mice (**Figure 4A** and **Table 1**). Notably, IL-13 was decreased compared to the control (**Figure 4A** and **Table 1**). Cytokines, such as granulocyte-macrophage (GM-CSF), INFγ, IL-1α, IL-2, IL-3, IL-4, IL-5, IL-10, IL-12, and IL-17, showed no obvious changes (**Figure 4A** and **Table 1**). Cluster analysis revealed that G-CSF, eotaxin, MIP-1β, MIP-1α, IL-6, TNFα, KC, MCP-1, IL-10, RANTES, GM-CSF, IL-12 (p40), INFγ, IL-1β, and IL-17 belonged to one cluster. IL-1α, IL-4, IL-12 (p70), IL-5, IL-3, IL-13, IL-2, and IL-9 belonged to another cluster.

Cytokines represent the strength of inflammation to some extent. The above results showed that mogroside II_E_ did not significantly influence the pathology of the pancreas from AP mice (**Figure 3E**). Therefore, it may show little effect on the level of cytokines. Mogroside II_E_ did not influence the reported inflammatory mediators in AP, including IL-1β, TNFα, IL-6, MCP-1, eotaxin, or RANTES. Other rarely reported cytokines, G-CSF and IL-13 were not changed significantly by cerulein + LPS +Mogroside II_E_ administration. Short and long term treatment of mogroside II_E_ did not alter the release of most cytokines. However, mogroside II_E_ decreased the level of IL-9 significantly (**Figure 4A** and **Table 1**).

Taken together, mogroside II_E_ specifically inhibited IL-9 release without affecting the release of many known AP-related cytokines, which suggests that mogroside II_E_ had no significant impact on the inflammation in AP.

### Mogroside II_E_ Inhibition of Digestive Enzymes Is Dependent on IL-9 in AP Mouse

Mogroside II_E_ decreased the activity of pancreatic trypsin, serum lipase and amylase in AP. We next investigated the mechanism underlying mogroside II_E_ mediated digestive enzyme blockage, especially trypsinogen activation inhibition. IL-9 increased significantly in AP and its release was selectively inhibited under mogroside II_E_ treatment (both short and long term). Previous report showed that cytokine could activate trypsinogen or induce digestive enzyme (Xu et al., 2014; Liu et al., 2017). Therefore, we hypothesized that mogroside II_E_-mediated digestive enzyme (trypsin, lipase and amylase) deactivation was related to IL-9. We performed a recovery experiment to investigate this hypothesis. IL-9 protein was injected into mogroside II_E_-treated AP mice, and the activities of related digestive enzymes were detected.

The results showed that trypsin and cathepsin B activation in pancreatic tissue decreased in the presence of mogroside II_E_, but this phenomenon was reversed by IL-9 injection (**Figures 4B, C**). Serum lipase and serum amylase, which were reduced by mogroside II_E_ in AP mice, increased after IL-9 injection (**Figures 4D, E**). These results indicate that mogroside II_E_ inhibits digestive enzymes in an IL-9-dependent manner in AP mice.

### Mogroside II_E_ Inhibits the IL-9/IL-9R/Calcium Overload Axis to Decrease the Activity of Digestive Enzymes in AP

**Figure 5:**
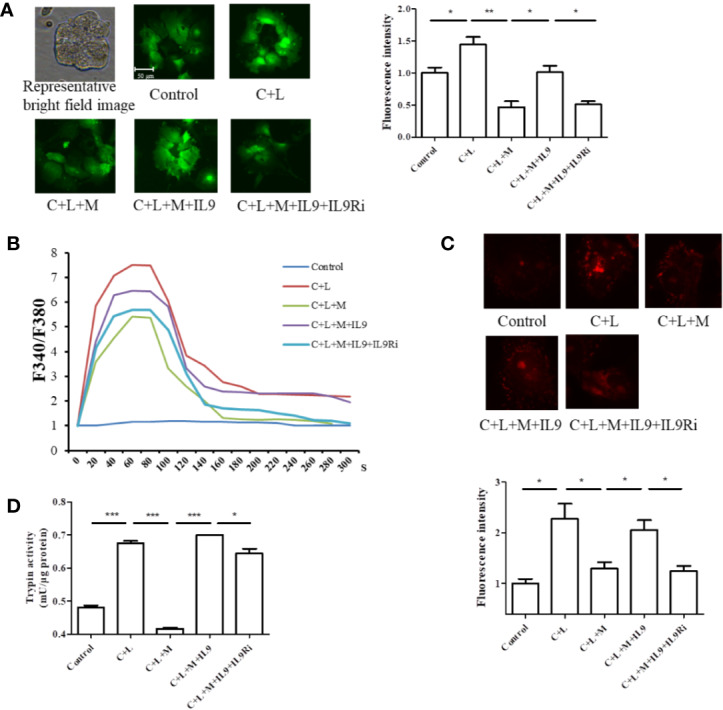
Mogroside II_E_ inhibits the IL-9/IL-9R/calcium overload axis to decrease the activity of digestive enzymes in AP. Primary pancreatic acinar cells were isolated from C57/BL6 mice. Cells were treated with solvent (Control), cerulein (200 nM) + LPS (10 ng/ml) (C + L), cerulean + LPS + Mogroside II_E_ (20 μM) (C + L + M), cerulean + LPS + Mogroside II_E_ + IL-9 (10 ng/ml) (C + L + M + IL9), cerulean + LPS + Mogroside IIE + IL-9 + IL-9R antibody (2.5 μg/ml) (C + L + M + IL9 + IL9Ri) for 6 h. **(A)** Cells were incubated with Furo-4 AM, and cells were imaged with fluorescence microscope. Fluorescence intensity was quantified using ImageJ software. Graph represents means ± SD from three independent experiments. ^*^P < 0.05, ^**^P < 0.001. **(B)** Primary acinar cells were loaded with Fura-2AM, and the indicated compounds were added. Fluorescence was detected dynamically with spectrophotometer. **(C)** The trypsin fluorescent substrate Rhodamine 110, bis-(CBZ-L-isoleucyl-L-prolyl-L-arginine amide) (Sigma) was added to cells after the indicated treatments. Cells were imaged with microscopy. The fluorescence intensity reflecting trypsin activity was quantified using ImageJ software. Graph represents means ± SD from three independent experiments. ^*^P < 0.05. **(D)** Cells were lysed and subjected to trypsin activity analyses. Graph represents means ± SD from three independent experiments. ^*^P < 0.05, ^***^P < 0.001.

Calcium overload can trigger cathepsin B followed by trypsinogen activation (Xiao et al., 2016). Therefore, cytosolic calcium was measured. Cells were stained with the calcium-specific dye fluro-4 AM or fura-2AM, and fluorescence was detected using a fluorescence microscope or spectrophotometer (**Figures 5A, B**). Cerulein plus LPS treatment induced cytosolic calcium rise, which was inhibited at the presence of mogroside II_E_ (**Figure 5A**). IL-9 reversed the effect of mogroside II_E_ on cytosolic calcium (**Figure 5A**). Notably, mogroside II_E_ reduced peak calcium amplitude and the calcium plateau (**Figure 5B**). IL-9 binds to IL-9 receptor (IL-9R) to exert its function (Chakraborty et al., 2019). IL-9R is known to be present in pancreatic acinar cells which was validated in our study (**Figure S4**). Therefore, we examined whether IL-9R was involved in the effect of IL-9 on cytosolic calcium. Using the IL-9R antibody it was found that the IL-9-induced cytosolic calcium overload was inhibited after IL-9R blockade (**Figures 5A, B**). The calcium plateau and amplitude regulated by IL-9 were also altered by IL-9R blockage (**Figure 5B**). Besides, the IL-9R antibody supressed trypsinogen activation (**Figures 5C, D**).

Taken together, mogroside II_E_ suppressed the IL-9/IL-9R/calcium overload/trypsinogen activation axis in AP.

### Mogroside II_E_ Inhibits the IL-9/IL-9R/Impaired Autophagy Axis to Decrease the Activity of Digestive Enzymes in AP

**Figure 6:**
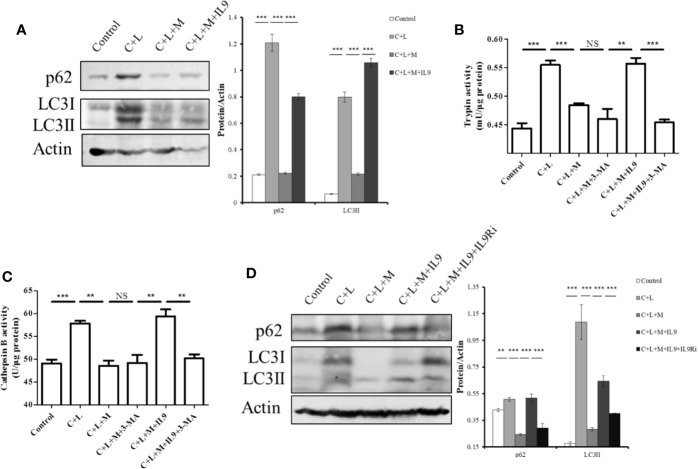
Mogroside II_E_ inhibits digestive enzymes *via* IL-9/impaired autophagy pathway. Primary pancreatic acinar cells under the indicated treatments were lysed, and cell lysates were subjected to Western blotting with anti-p62, anti-LC3, anti-β-actin antibodies **(A**, **D)**. The graph beside the blot represents means grey values ± SD quantified in ImageJ from three independent experiments. ^**^P < 0.001, ^***^P < 0.001. Cells under the indicated treatments were lysed and subjected to trypsin **(B)** and cathepsin B **(C)** activity assays. The graph represents means ± SD from three independent experiments. NS, nonsignificant, ^**^P < 0.001, ^***^P < 0.001.

Impaired autophagy promotes trypsinogen activation in AP (Mareninova et al., 2009; Bootman et al., 2018). Therefore, we investigated the effect of IL-9 on autophagy in AP. Cerulein plus LPS increased the protein level of p62 and LC3II, which indicated impaired autophagy upregulation (**Figure 6A**). Mogroside II_E_ decreased the protein level of p62 and LC3II in AP, which was reversed by IL-9 (**Figure 6A**). Meanwhile, we found that the recovery of the activity of trypsin and cathepsin B by IL-9 was inhibited under treatment with the autophagy inhibitor 3-MA (**Figures 6B, C**). IL-9R blockade inhibited IL-9 function in autophagy (**Figure 6D**).

Taken together, mogroside II_E_ blocked the IL-9/IL-9R/impaired autophagy pathway, which inhibited trypsinogen activation in AP.

### Mogroside II_E_ Targeting of IL-9 in AP May Be Partially From Th9 Cells

**Figure 7:**
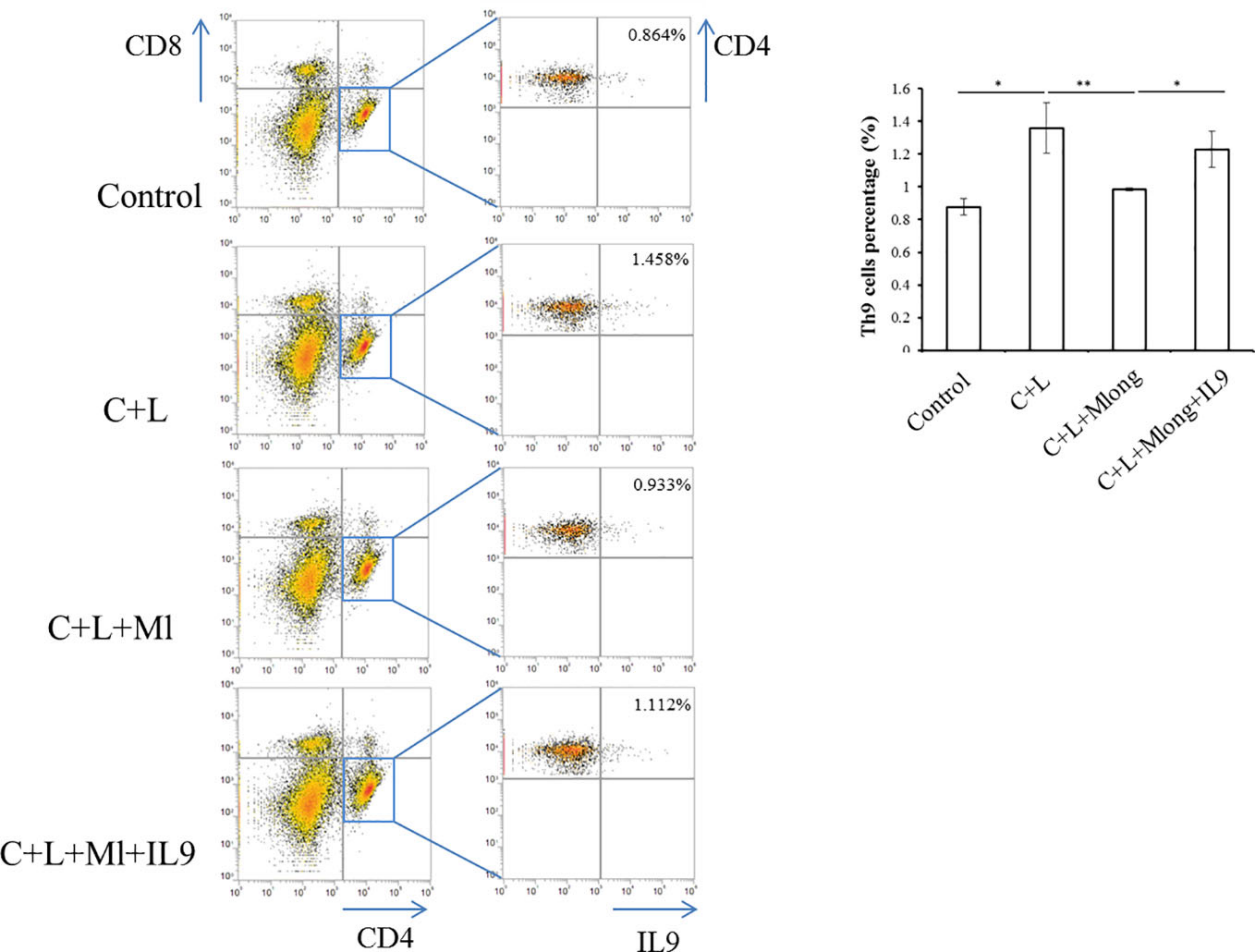
IL-9 targeted by mogroside II_E_ may be released from Th9 cells. Mice were divided into four groups (n = 3): Control group, C + L group, C + L + Mlong group, C + L + M + IL9 group. Th9 cells in spleen were counted using a flow cytometer with specific antibodies. ^*^P < 0.05, ^**^P < 0.001. The representative percentages of Th9 cells in spleen from mice with the indicated treatments were labeled. Graph represents means ± SD from three independent experiments.

IL-9 was released primarily from T cells, including Th9 (Chakraborty et al., 2019). To determine the source of IL-9 regulated by mogroside II_E_, we analyzed T cell subsets in spleen. Th9 cells were quantified using flow cytometry and specific antibodies. The results showed that the amount of Th9 cells increased in cerulein plus LPS-treated mice (**Figure 7**). Mogroside II_E_ reduced Th9 cells in AP, which was reversed by IL-9 (**Figure 7**). Notably, the detected Th9 cells counts a very small part of the Th cells in AP. These results indicated that IL-9 which worked as the downstream mediator of mogroside II_E_, may be partially released from Th9 cells.

## Discussion

Our study focused on the biological activity of mogroside II_E_. Mogroside II_E_ decreased the cerulein plus LPS-induced activity of trypsin and cathepsin B in the pancreatic acinar cell line AR42J and primary acinar cells. Mogroside II_E_ treatment decreased the level of serum lipase and serum amylase in mice injected with cerulein plus LPS. Multi-cytokine array and HE staining results showed that inflammation was not significantly changed in AP mice after mogroside II_E_ treatment, but the level of IL-9 in AP mice was selectively decreased. Mogroside II_E_ inhibition of digestive enzymes in AP was related to IL-9.

Mogroside II_E_ is primarily present in the unripe fruit of *S. grosvenorii* (Swingle), and it is the predominant saponin component (Wang et al., 2014). The representative digestive inhibitor, somatostatin and ulinastatin, are used to treat AP patients (Moggia et al., 2017). However, trypsin suppressors are not well studied. Our research showed that mogroside II_E_ may be a trypsin suppressor, which is the first report on the biological function of mogroside II_E_.

Mogroside III_E_ and mogroside V are two other saponin components of the fruit of *S. grosvenorii* (Swingle) and have similar molecular structures to mogroside II_E_. These two compounds protect mice from LPS-induced acute lung injury (Shi et al., 2014; Tao et al., 2017a). Therefore, mogroside II_E_ may be used to ameliorate lung complications in AP. Due to the structural similarity, mogroside III_E_ and mogroside V may also inhibit IL-9 pathway and trypsinogen activation. Notably, the trypsin inhibition effect exerted by mogroside II_E_ was small but significant. Therefore, structural modifications of mogroside II_E_ may be performed to find a better trypsin inhibitor.

We investigated the roles of mogroside II_E_ in AP further. However, this compound did not significantly ameliorate the pathology of the pancreas, including inflammatory cell infiltration. Consistently, mogroside II_E_ did not significantly influence cytokine release under cerulein plus LPS treatment, except KC, MIP-1 and IL-9. Premature trypsin and NFкB activation occur in parallel to promote AP (Sah et al., 2012). Here, mogroside II_E_ inhibited trypsin *in vitro* and *in vivo*. Therefore, combined mogroside II_E_ and NFкB inhibitors may be useful for AP therapy.

The cytokine array in our study illustrated that many cytokines were involved in AP. Apart from the classic pro-inflammatory cytokines in AP, G-CSF, eotaxin, IL-9, and RANTES, which are rarely reported to be associated with AP, were upregulated in an AP experimental model. The known anti-inflammatory IL-10 showed minor changes during AP, which may be due to it achieving peak value before the detection. Notably, IL-13 levels decreased in the AP model. Although, IL-13 may work as an anti-inflammatory mediator, clinical analysis showed that IL-13 upregulation was related to more severe AP (Rodriguez-Nicolas et al., 2018). The actual role of IL-13 must be investigated further.

According to our array results, IL-9 significantly increased in AP and mogroside II_E_ specifically suppressed the IL-9 release induced by cerulein plus LPS. Although mogroside II_E_ inhibited cerulein plus LPS-induced trypsinogen activation *via* IL-9, other factors may be involved in this process. It's needed to be mentioned here that IFNγ which has been reported to induce trypsinogen activation (Liu et al., 2017) didn't change significantly under mogroside II_E_ treatment.

IL-9 was previously found to increase cytosolic calcium in interstitial cells (Huang et al., 2016), which was also observed in pancreatic acinar cells in our research. IL-9 is increased in T cells lacking Atg 3 or Atg 5 compared to the control (Rivera Vargas et al., 2017). Atg 3 and Atg 5 are the key mediators for autophagy flux induction, which suggests that autophagy negatively regulated IL-9. Our study suggested that IL-9 induced impaired autophagy, which was not reported previously.

IL-9 is primarily released from T cells, including Th2, Th9 and Th17 cells. Other cells, such as mast cells, also secrete IL-9 (Chakraborty et al., 2019). We found that IL-9, which mediated trypsinogen activation and was down-regulated by mogroside II_E_, can be secreted by other cells. Notably, a previous report showed that Th9 cells work in allergic and asthmatic conditions (Karagiannis and Wilhelm, 2017). Our findings have uncovered a new physiological role of Th9 cells, which is mediating trypsinogen activation in the pancreas during acute pancreatitis.

### Summary Conclusion

**Figure 8:**
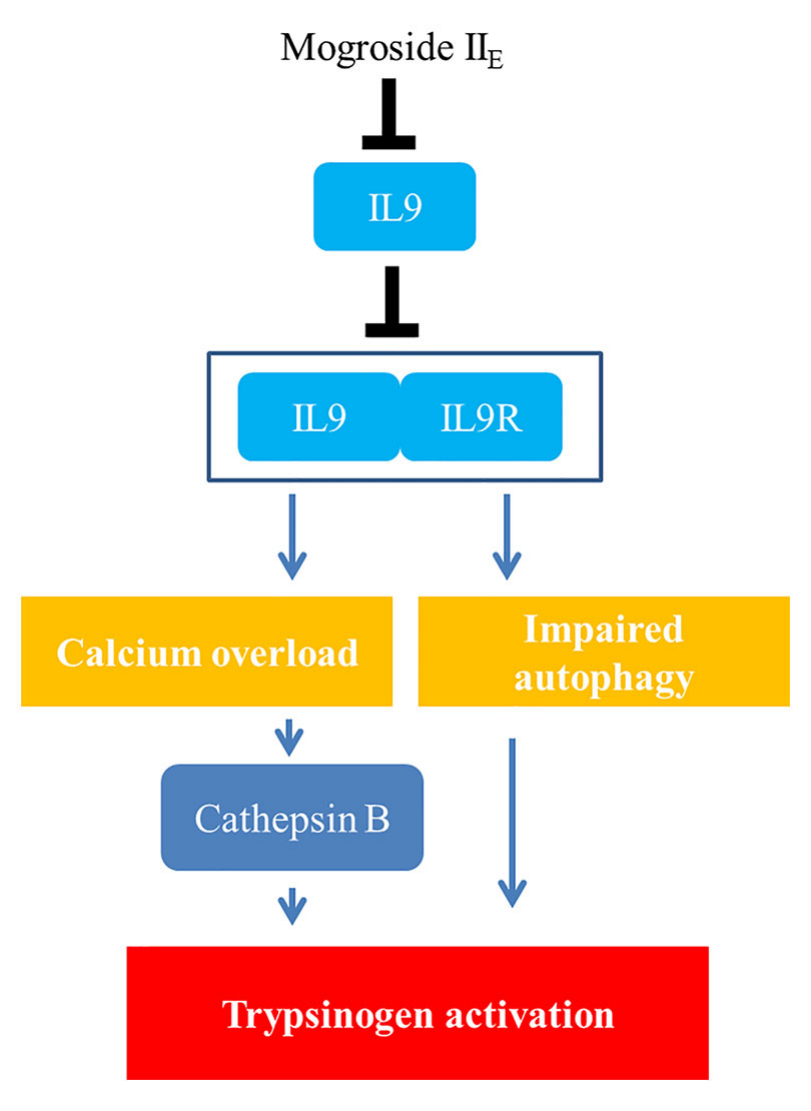
Diagram of the purported mechanism of mogroside II_E_ activity on pancreatic cells.

Taken together, the signalling network in this manuscript is summarized as mogroside II_E_ inhibited the IL-9/IL-9R/calcium overload/cathepsin B activation/trypsinogen activation pathway. Meanwhile, mogroside II_E_ suppressed IL-9/IL-9R/calcium overload/impaired autophagy/trypsinogen activation signalling transduction. The relative mechanism is summarized in **Figure 8**.

## Data Availability Statement

All datasets generated for this study are included in the article/**Supplementary Material**.

## Ethics Statement

The animal study was reviewed and approved by Ethical Committee on Animal Experiments at Guilin Medical University.

## Author Contributions

Participated in research design: JX, JJ, DL. Performed experiments: JX, KH, HL, ZX, JZ. Contributed new reagents or analytic tools: DL. Performed data analysis: JX, KH, HL. Wrote or contributed to the writing of the manuscript: JX, JJ. Obtained the funding: JX, JJ, DL.

## Funding

This study was supported in part by the National Natural Science Foundation of China (No. 81800576, 81960127, 21562009), the recruitment program for the Affiliated Hospital of Guilin Medical University, and the Science and Technology Planned Project in Guilin (20170303, 20190206-1). The present study was also supported by the Lijiang Scholar Award in Guilin (2017-004), the High Level of Innovation Team and Outstanding Scholars Program in Colleges and Universities in Guangxi (2017-38-08), and the Guangxi Distinguished Experts Special Fund (2019-13-12).

## Conflict of Interest

The authors declare that the research was conducted in the absence of any commercial or financial relationships that could be construed as a potential conflict of interest.

## Supplementary Material

Figure S1 Serum lipase and serum amylase increased in a time-dependent manner in acute pancreatitis animal model. C57BL/6 mice were intraperitoneally injected with saline (S) or cerulein plus LPS (C+L) seven times at one-hour intervals. Mice were sacrificed at the indicated hours after the final injection. Serum lipase **(A)** and amylase **(B)** were measured. C+L vs. S at each time point, ^*^*P* < 0.05, ^**^*P* < 0.01, ^***^*P* < 0.001.

Figure S2 Mogroside II_E_ did not influence serum lipase and amylase at 1 h **(A**, **B)** or 3 h **(C**, **D)** after the final injection. C+L vs. Control, ^***^*P* < 0.001; C+L+M vs. C+L, NS, nonsignificant.

Figure S3 Pathological scores of pancreas. Sections of pancreas collected were scored as described in Methods. Means ± SD of the scores for edema **(A)**, neutrophil infiltration **(B)** and necrosis **(C)** as well as combined (total = edema + neutrophil infiltration + necrosis) **(D)** are presented. NS nonsignificant.

Figure S4 IL-9R is present in pancreatic cells. AR42J cells and primary acinar cells were lysed and subjected to Western blotting analysis using the indicated antibodies.
