# Supplementary figures and images for: Mogroside IIE Inhibits Digestive Enzymes via Suppression of Interleukin 9/Interleukin 9 Receptor Signalling in Acute Pancreatitis
Source: Front Pharmacol. 2020 Jun 10;11:859. doi: 10.3389/fphar.2020.00859 (PMC7298197; doi:10.3389/fphar.2020.00859)

Figure S1

A

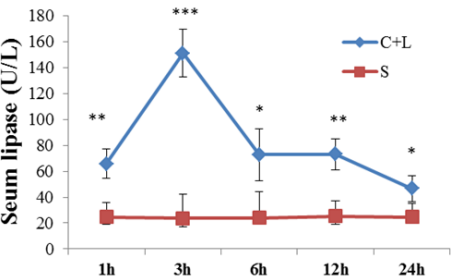

B

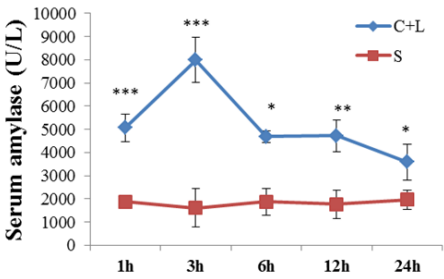

Supplement: Figure S1 — Serum lipase and serum amylase increased in a time-dependent manner in acute pancreatitis animal model. C57BL/6 mice were intraperitoneally injected with saline (S) or cerulein plus LPS (C+L) seven times at one-hour intervals. Mice were sacrificed at the indicated hours after the final injection. Serum lipase (A) and amylase (B) were measured. C+L vs. S at each time point, *P < 0.05, **P < 0.01, ***P < 0.001. [file Image_1.pdf]

Figure S2

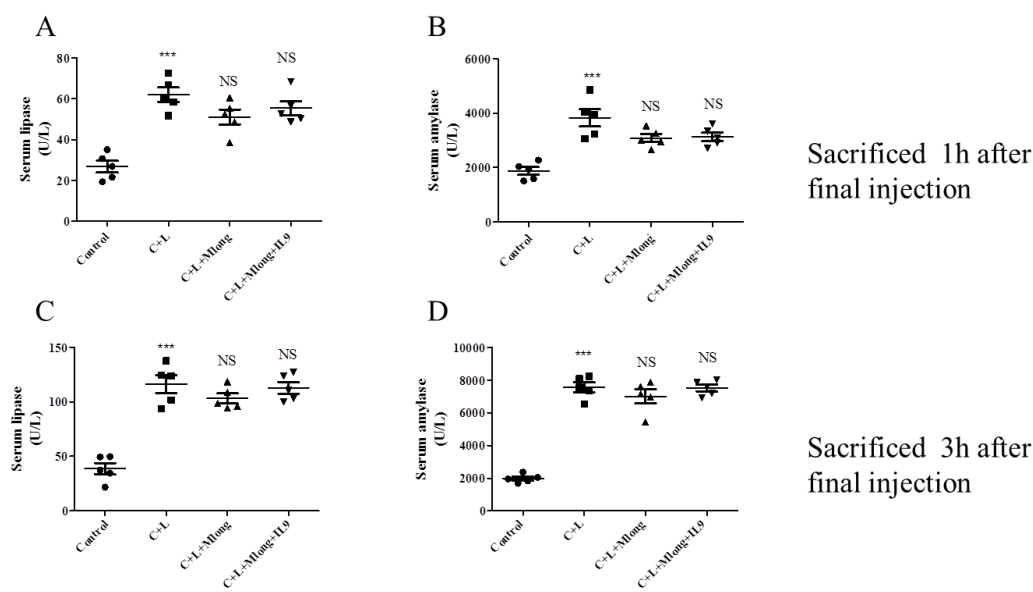

Supplement: Figure S2 — Mogroside IIE did not influence serum lipase and amylase at 1 h (A, B) or 3 h (C, D) after the final injection. C+L vs. Control, ***P < 0.001; C+L+M vs. C+L, NS, nonsignificant. [file Image_2.pdf]

Figure S3

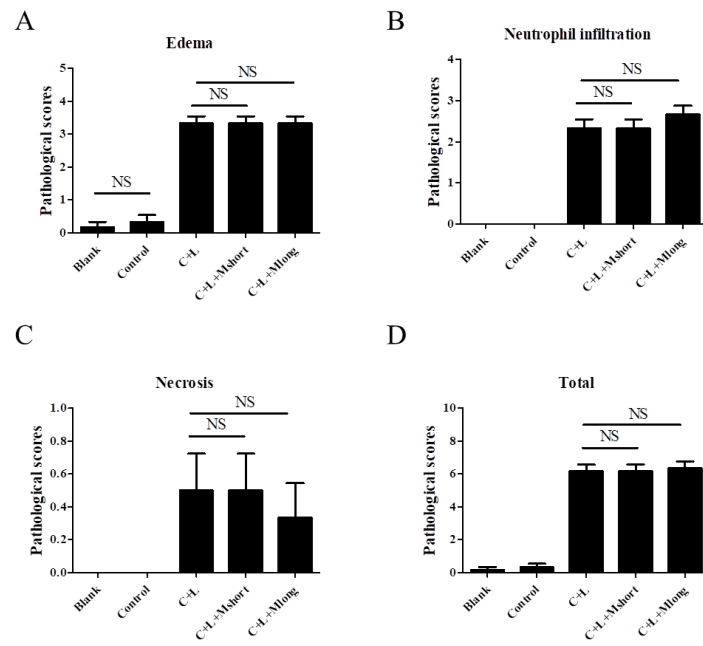

Supplement: Figure S3 — Pathological scores of pancreas. Sections of pancreas collected were scored as described in Methods. Means ± SD of the scores for edema (A), neutrophil infiltration (B) and necrosis (C) as well as combined (total = edema + neutrophil infiltration + necrosis) (D) are presented. NS nonsignificant. [file Image_3.pdf]

Figure S4

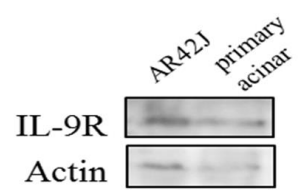

Supplement: Figure S4 — IL-9R is present in pancreatic cells. AR42J cells and primary acinar cells were lysed and subjected to Western blotting analysis using the indicated antibodies. [file Image_4.pdf]
